# A Marfan syndrome gene expression phenotype in cultured skin fibroblasts

**DOI:** 10.1186/1471-2164-8-319

**Published:** 2007-09-12

**Authors:** Zizhen Yao, Jochen C Jaeger, Walter L Ruzzo, Cecile Z Morale, Mary Emond, Uta Francke, Dianna M Milewicz, Stephen M Schwartz, Eileen R Mulvihill

**Affiliations:** 1 Department of Computer Science and Engineering, University of Washington, Seattle, Washington 98195, USA; 2 Department of Genome Sciences, University of Washington, Seattle, Washington 98195, USA; 3 Department of Pathology, University of Washington, Seattle, Washington 98195, USA; 4 Department of Biostatistics, University of Washington, Seattle Washington 98195, USA; 5 Departments of Genetics and Pediatrics, Stanford University, Stanford, CA 94305-5323, USA; 6 University of Texas Medical School at Houston, 6431 Fannin, MSB 1.614, Houston, TX 77030, USA; 7 Hamilton Robotics, Via Crusch 8, Bonaduz, Switzerland; 8 Trubion Pharmaceuticals Inc., Seattle, Washington 98121, USA; 9 PO Box 33, Villanueva, NM 87583, USA

## Abstract

**Background:**

Marfan syndrome (MFS) is a heritable connective tissue disorder caused by mutations in the fibrillin-1 gene. This syndrome constitutes a significant identifiable subtype of aortic aneurysmal disease, accounting for over 5% of ascending and thoracic aortic aneurysms.

**Results:**

We used spotted membrane DNA macroarrays to identify genes whose altered expression levels may contribute to the phenotype of the disease. Our analysis of 4132 genes identified a subset with significant expression differences between skin fibroblast cultures from unaffected controls versus cultures from affected individuals with known fibrillin-1 mutations. Subsequently, 10 genes were chosen for validation by quantitative RT-PCR.

**Conclusion:**

Differential expression of many of the validated genes was associated with MFS samples when an additional group of unaffected and MFS affected subjects were analyzed (p-value < 3 × 10^-6 ^under the null hypothesis that expression levels in cultured fibroblasts are unaffected by MFS status). An unexpected observation was the range of individual gene expression. In unaffected control subjects, expression ranges exceeding 10 fold were seen in many of the genes selected for qRT-PCR validation. The variation in expression in the MFS affected subjects was even greater.

## Background

Aneurysm and dissection are major diseases of the aorta and are often asymptomatic until a life-threatening event like ischemic organ damage or rupture occurs. Marfan Syndrome (MFS) is a diverse yet clinically recognized subgroup of people at risk for aneurysm, including dissecting aneurysm, and constitutes a significant fraction (estimated at 5–7.5% [1,2]) of all individuals with ascending and thoracic aortic aneurysmal disease. MFS incidence is estimated to be 1 in 5–10,000 [3]. Our long-term goal is to develop an assay that will identify people at risk for aneurysm before the disease process has reached an advanced state. This report is a small step in that direction.

In this study, we focus on individuals diagnosed with Marfan syndrome. The prevalence of MFS combined with its clinical recognition makes it an excellent model system for studies on aneurysmal disease. MFS is an autosomal dominant heritable disorder caused by mutations in the fibrillin-1 (*FBN1*) gene [4,5], with more than 500 unique mutations identified [6]. *FBN1 *mutations show a high degree of penetrance but considerable inter- and intra-familial variability in their phenotype [3]. The variable penetrance suggests that environmental factors and/or disease modifying genes also contribute to the phenotype. Neonatal MFS correlates to mutations within exons 24–32 and MFS defined by mutations in exons 59–65 carry a reduced risk of aortic pathology. Large-scale comparisons between MFS individuals with premature termination mutations and cysteine substitutions in *FBN1 *revealed significant differences in ocular, skeletal and hypermobility features but no difference in the frequency of ascending aortic aneurysm [7,8]. Apart from these observations, determining the nature of the mutation (a time and labor intensive process) does not improve prediction of the severity of the disease, the risk of aneurysm development or of its progression [7,9]. These limited genotype-phenotype correlations suggest that genes other than *FBN1 *may significantly influence the phenotype, and their identification may lead to a more informative test of risk.

Fibroblasts are not smooth muscle cells. However, in culture they display a stable phenotype with stress fibers composed of cytoplasmic actins and a splice variant of cellular fibronectin [10]. The increased mechanical stress on dermal fibroblasts seeded at low density produces a cell culture population consisting of 70–80% myofibroblasts. The term "myofibroblast" was proposed over 30 years ago to describe the fibroblasts that appeared in granulation tissue at the sight of open wounds [11]. Recently, it has been recognized that Thy-1 surface expression defines a subpopulation of fibroblasts capable of differentiating into myofibroblasts [12]. We can detect Thy-1 expression in both affected and unaffected skin fibroblasts by array and have confirmed that observation by quantitative real time polymerase chain reaction (qRT-PCR, data not shown). Thus, the skin cultures we used were "myofibroblast" like.

In the last several years, use of DNA microarrays to analyze gene expression has emerged as a promising technology for disease classification and prognosis and for identification of genes that could be potential causes, bio-markers or drug targets [13-18]. However, there are limits to the sensitivity of microarrays for detecting genes expressed at low levels as well as additional confounding problems associated with arrays [19-23]. Consequently, in common with most recent studies [20,24], we go beyond mere classification by independently validating expression levels using quantitative qRT-PCR and validating the results in a second population.

In the present study, we used total RNA in oligo dT primed cDNA reactions to identify an expression phenotype associated with the MFS genotype in cultured skin fibroblasts. Our results show a clearly recognizable expression phenotype in cultured fibroblasts. We of course do not expect exactly the same expression phenotype in aortic smooth muscle cells, but we do expect some overlap in the perturbed pathways, as they share the same root cause. Some of the identified genes, including elastin and several collagens, are obvious targets for roles in the development and maintenance of the extracellular matrix environment and cell-matrix contacts. Additional genes validated by qRT-PCR analysis, including the vitamin D receptor, programmed cell death-10 and the LIM domain only 7, may represent genes that will provide new insight into the disease process. Our ability to detect a MFS expression phenotype in cultured fibroblasts provides both a simple method for large-scale screening and a basis for mechanistic studies of the genes identified as differentially expressed.

## Results

**Figure 1:**
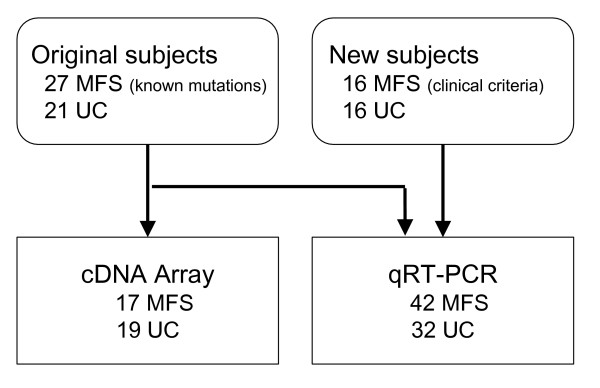
**Overview of the experimental design**. We used spotted membrane DNA arrays to characterize gene expression from MFS samples with known *FBN1 *mutations and from unaffected control samples. Following analysis of both groups, we selected a set of genes for validation by qRT-PCR, using a majority of the original samples. A new population of 16 probable MFS, all with an aortic phenotype, and 16 UC samples were used to test independently whether the selected genes were differentially expressed between the two groups. See Table 1 for details about subjects included in each experiment.

An overview of our experimental design is shown in Figure 1. In brief, we analyzed total RNA from 36 subjects by spotted membrane DNA macroarray. We used Research Genetics spotted cDNA membrane arrays to analyze gene expression in fibroblast cultures, 17 with characterized mutations in the *FBN1 *gene (7 missense, all cysteine substitutions, 9 nonsense and 1 multi-exon deletion; 14 of 17 had an aortic phenotype) and 19 unaffected controls (details in Table 1). We selected differentially expressed genes between MFS and unaffected samples based on estimated False Discovery Rate (FDR)[25]. We calculated the 4132 p-values for the two-sample t-tests associated with the expression differences between affected and control for each gene on the array. At a q-value (estimated FDR) threshold of 0.001, 283 genes (265 with identifiable unique Unigene ID numbers) out of a total of 4132 are selected as differentially expressed in MFS vs. unaffected. The top ranking genes based on ratio are listed in Table 2 [see Additional File 1 for the entire list]. At the same q-value threshold, we found 175 differentially expressed genes (165 with unique Unigene ID) between missense and unaffected samples, and 111 differentially expressed genes (106 with unique Unigene ID) between nonsense and unaffected samples. The q-values are only approximate, since their validity depends on various assumptions such as normality of the expression levels within groups. To globally assess the reliability of these test results while making fewer statistical assumptions, we performed a permutation test (see Methods) to determine if the large number of significant genes we found could easily have arisen by chance. Of 50,000 random permutations for MFS and unaffected samples, *no *permutations have more than 283 differentially expressed genes, making the array results highly statistically significant, with an empirical p-value smaller than 0.00002. Furthermore, the only permutations exhibiting a substantial number of significant genes were those permutations that happened to separate most of the MFS subjects from most of the unaffected controls. Over forty five thousand permutations showed *no *significant genes, and the average number called significant was only 0.45, making it very likely that our estimated false discovery rate of 0.001 is conservative. [See Additional File 1 for the complete 4132-gene dataset.]

**Table 1:** 

	**MFS Affected (known mutations)**								**Unaffected Controls (UC)**					
	**Subject**		**Sex**	**Age**	**Mutation**	**Platform**		**Ref.**	**Subject**		**Sex**	**Age**	**Platform**	

1	FB	969	F	53	C628X	A	T	30	UC	1	F	74		T
2	FB	992	M	49	R861X	A	T	30	UC	2	F	34		T
3	GD	1051	F	45	PTC ex 24	A	T	DMUP	UC	3	F	34	A	T
4	FB	1234	F	34	N1157X	A	T	30	UC	4	F	34	A	T
5	FB	773	F	48	D1191X	A	T	30,61	UC	5	F	38	A	T
6	FB	857	M	16	R1192X		T	30	UC	6	F	35	A	T
7	FB	751	F	54	L1412X		T	30	UC	7	F	15	A	T
8	FB	997	F	48	R1523X		T	30	UC	8	F	11	A	
9	FB	1286	F	33	R2057X	A	T	30	UC	9	F	5	A	
10	60	FR	M	7	PTC ex 63	A	T	DMUP	UC	10	F	25	A	T
11	GD	021	M	17	PTC ex 63	A	T	DMUP	UC	11	F	49	A	
12	GD	032	F	9	PTC ex 64	A		DMUP	UC	12	F	40	A	
13	FB	836	F	31	C832Y	A	T	8,61	UC	13	M	72	A	T
14	FB	837	M	10	C832Y	A	T	8,61	UC	14	M	49	A	T
15	FB	783	F	33	C1117Y	A	T	61	UC	15	M	36	A	T
16	FB	984	M	22	C1171R		T	8	UC	16	M	43	A	T
17	FB	1069	F	4	C1326R		T	8	UC	17	M	35	A	T
18	FB	1040	F	29	C1361Y	A	T	8	UC	18	M	15	A	T
19	FB	882	F	27	C1402W		T	8	UC	19	M	71	A	T
20	FB	881	M	24	C1589F	A	T	61	UC	20	M	14	A	T
21	FB	1627	M	34	C2038Y	A	T	UFUP	UC	21	M	50	A	
22	FB	829	M	21	C2053F		T	62						
23	FB	1211	M	20	C2111R	A	T	UFUP						
24	FB	1359	M	28	C2686F		T	62						
25	FB	890	F	4mo	del ex 44-46	A	T	63						
26	FB	774	F	40	del ex 42-43		T	63						
27	FB	970	M	38	del ex 54		T	63						

	Average Age 29								Average Age 37					

	**Additional MFS Affected (unknown mutations)**								**Additional Unaffected Controls (UC)**					

	Subject		Sex	Age		Platform			Subject		Sex	Age	Platform	

28	MFS	1	M	37	AF		T		UC	22	M	25		T
29	MFS	3	M	25	AF		T		UC	23	F	56		T
30	MFS	4	F	45	AF		T		UC	24	M	39		T
31	MFS	5	F	68	AF		T		UC	25	F	38		T
32	MFS	6	M	48	AF		T		UC	26	F	59		T
33	MFS	7	F	29	AF		T		UC	27	M	30		T
34	MFS	8	M	44	AF		T		UC	28	M	32		T
35	MFS	9	F	55	AF		T		UC	29	M	55		T
36	MFS	10	F	17	AF		T		UC	30	M	43		T
37	MFS	11	F	42	AF		T		UC	31	F	58		T
38	MFS	12	M	32	AF		T		UC	32	F	52		T
39	MFS	13	F	47	AF		T		UC	33	F	23		T
40	MFS	14	M	17	AF		T		UC	34	F	55		T
41	MFS	17	M	53	AF		T		UC	35	M	38		T
42	MFS	18	M	38	AF		T		UC	366	F	36		T
43	MFS	20	M	46	AF		T		UC	37	F	41		T
	Average Age 40								Average Age 43					
					Total:	17		42				Total:	19	32

Twenty-one previously described MFS affected subjects with identified FBN1 mutations [8,30,61-63] and 6 additional affected subjects (UFUP, U. Francke unpublished; DMUP, D. Milewicz unpublished) representing 25 different mutations in the *FBN1 *gene were studied. Sixteen additional MFS affected subjects (AF) volunteered. Their clinical phenotype was consistent with MFS as determined by a group of examining physicians at the National Marfan Foundation annual conference. UC 1–37 are unaffected control subjects recruited from dermatology clinics. The total RNA from primary skin fibroblast cultures was characterized by two different technology platforms that detect gene expression differences: DNA array (A) and quantitative RT-PCR (T). The thirty-two additional subjects ("new subjects") were added for the validation phase of the study.

**Table 2:** Top 15 genes identified by ratio from the array dataset

**UG cluster ID**	**SwissProt Accession**	**Gene name**	**MFS NI**	**UC NI**	**Ratio MFS/UC**	**q-value**
Hs.146688	PTGES:O14684	Prostaglandin E synthase	185	108	1.71	1.36E-03
Hs.90303	TSC2:P49815	Tuberous sclerosis 2	60	37	1.64	1.26E-04
Hs.1872	PCK1:P35558	Phosphoenolpyruvate carboxykinase 1	249	157	1.59	4.11E-03
Hs.310545	SYT1:P21579	Synaptotagmin I	109	69	1.57	2.60E-03
Hs.144700	EFNB1:P98172	Ephrin-B1	296	191	1.56	4.84E-03
Hs.334534	GNS:P15586	Glucosamine (N-acetyl)-6-sulfatase	173	112	1.54	5.23E-05
Hs.386283	ADAM12:O43184	Meltrin L	44	73	1.65^-1^	5.08E-09
Hs.516646	CREB1:P16220	CAMP responsive element BP 1	193	339	1.76^-1^	1.96E-05
Hs.489142	COL1A2:P08123	Collagen, type I, alpha 2	3663	6582	1.80^-1^	4.48E-06
Hs.146447	FBN1:P35555	Fibrillin 1	124	233	1.89^-1^	2.52E-10
Hs.250581	SMARCD2:Q92925	SWI/SNF D2	1532	2963	1.93^-1^	5.99E-06
Hs.432862	MARCH-VI:O60337	RING-CH protein VI	2091	4245	2.03^-1^	4.97E-09
Hs.55967	SHOX2:O60902	Short stature homeobox 2	58	137	2.38^-1^	1.13E-12
Hs.443625	COL3A1:P02461	Collagen, type III, alpha 1	638	1538	2.41^-1^	2.06E-04
Hs.252418	ELN:P15502	Elastin	35	117	3.34^-1^	2.19E-08

Unigene ID identifies significant, differentially expressed genes based on expression ratio criteria. The top 6 down regulated and 9 up regulated genes are included. The normalized intensity (NI) from the phosphorimager scan is listed to give a sense of the expression range. The q values are computed based on t test p values. In this and subsequent tables, we show all ratios less than 1 as reciprocals. The entire 4132 gene list has been deposited into GEO (the Gene Expression Omnibus), accession number GSE8759.

### Quantitative RT-PCR

**Figure 2:**
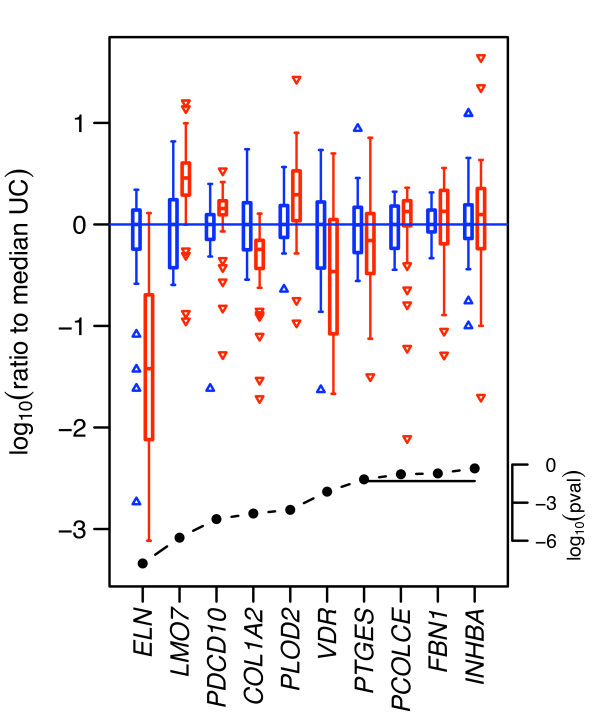
**Validation by quantitative qRT-PCR**. The figure presents a summary of the 10 genes selected for validation by qRT-PCR. Above each gene name are two "box-and-whisker" plots of expression levels for that gene across 32 unaffected control (UC) samples (left plot of each pair, blue, up-triangles) and 42 MFS affected samples (right, red, down-triangles). Vertical axis is log_10 _ratio of expression level to the median UC level. Each "box" shows the inter-quartile range (IQR), i.e., the range between the 25^th ^and 75^th ^percentiles of the log ratios; the horizontal line in each box is the 50th percentile (median). (Median log ratio for UC is always zero, by definition.) "Whiskers" (vertical lines) extend from each box to the most extreme values within 1.5 times the IQR from the box; in normally distributed data this would on average encompass 99% of the values. Triangles mark more extreme points. The lower curve shows log_10 _(p-value) for a Wilcoxon rank sum test of the null hypothesis that the UC and MFS distribution are identical; horizontal line marks the p = 0.05 significance level. 6 of 10 genes have p-values < 0.05 by this test. Most genes exhibit noticeably greater variability across the MFS samples than across UC samples, although the Wilcoxon test is not sensitive to this. To highlight one example, for Elastin (*ELN*), the middle 50% of the UC sample log ratios fall between -0.23 and +0.14 (i.e., the 25^th ^and 75^th ^percentiles of the values fall 1.70-fold below and 1.38-fold above the median, respectively), and all but 4 fall between -0.58 and +0.34 (4-fold below and 2.2-fold above median). In contrast, median Elastin level is 26 fold lower in MFS samples, only four MFS samples are above the UC median, and the null hypothesis has a p-value of 1.6 × 10^-8^.

We performed duplicate quantitative RT-PCR assays on total RNA from 74 subjects (Table 1) to measure the concentration of 12 mRNAs [see Additional file 2]. We chose 10 genes based on the degree of ratio change, small q-value and the availability of Applied Biosystems predetermined assay reagents (PDARs). We included two additional genes, *GUSB *and *TBP *as internal references. Behaviors of these genes in the array are summarized in Table 3. The qRT-PCR data for a given subject are generally positively correlated with the array results for the same 10 genes from the same RNA sample, with a mean Spearman (rank) correlation of 0.46 (0.35 among unaffected subjects, 0.52 among MFS subjects). Figure 2 summarizes qRT-PCR results for all 74 subjects, and Table 4 additionally summarizes qRT-PCR results for the array and new subjects separately.

**Table 3:** Data summary of genes selected for validation by qRT-PCR

**SwissProt Accession**	**Gene name**	**PDAR**	**MFS NI**	**UC NI**	**Ratio MFS/UC**	**q value**
*VDR:*P11473	vitamin D receptor	Hs00172113_m1	59	84	1.42^-1^	**3.94E-11**
*FBN1:*P35555	fibrillin 1	Hs00171791_m1	124	233	1.89^-1^	**2.52E-10**
*INHBA:*P08476	inhibin, beta A	Hs00170103_m1	72	117	1.61^-1^	**3.48E-10**
*ELN:*P15502	elastin	Hs00355783_m1	35	117	3.34^-1^	**2.19E-08**
*COL1A2:*P08123	collagen, type I, alpha 2	Hs00164099_m1	3663	6582	1.80^-1^	**4.48E-06**
*PCOLCE:*Q15113	procollagen C-endopeptidase	Hs00170179_m1	134	168	1.26^-1^	**2.99E-04**
*PLOD2:*O00469	lysine hydroxylase 2	Hs00168688_m1	92	75	1.22	**3.00E-04**
*PDCD10*Q9BUL8	programmed cell death 10	Hs00200578_m1	534	464	1.15	**1.29E-03**
*PTGES:*O14684	prostaglandin E synthase	Hs00610420_m1	185	108	1.72	**1.36E-03**
*LMO7:*Q8WWI1	LIM domain only 7	Hs00245600_m1	50	45	1.13	**5.07E-03**

We selected 10 genes for validation based on their performance on the array. Column headings are as in Table 2, except PDAR: Applied Biosystems predetermined assay reagent identifier.

**Table 4:** QRT-PCR validation and phenotype prediction

		**qRT-PCR results: MFS/UC ratio (Wilcoxon p-value)**					
		
**Gene symbol**	**Array ratio**	**Array subjects**		**New subjects**		**All subjects**	
*VDR*	1.42^-1^	**4.59**^-1^	**(9.5e-5)**	1.25	(8.1e-1)	**2.53**^-1^	**(7.3e-3)**
*FBN1*	1.89^-1^	**1.47**^-1^	(5.2e-2)	*2.71*	(3.3e-9)	N/A	
*INHBA*	1.61^-1^	2.20	(1.1e-1)	**1.06**^-1^	(6.7e-1)	1.05	(5.0e-1)
*ELN*	3.34^-1^	**12.53**^-1^	**(2.4e-3)**	**9.68**^-1^	**(2.3e-4)**	**15.04**^-1^	**(1.6e-8)**
*COL1A2*	1.80^-1^	**1.24**^-1^	(1.1e-1)	**2.41**^-1^	**(2.3e-8)**	**2.27**^-1^	**(1.4e-4)**
*PCOLCE*	1.26^-1^	**1.27**^-1^	(5.8e-2)	*2.02*	(4.6e-6)	N/A	
*PLOD2*	1.22	**3.01**	**(3.8e-4)**	**1.69**	**(8.2e-3)**	**1.85**	**(2.7e-4)**
*PDCD10*	1.15	**1.98**	**(1.9e-6)**	**1.65**	**(4.8e-3)**	**1.41**	**(5.0e-5)**
*PTGES*	1.72	*3.42*^-1^	(5.8e-4)	**1.88**	**(9.3e-3)**	1.68^-1^	(6.8e-2)
*LMO7*	1.13	**2.01**	**(1.0e-3)**	**4.67**	**(3.0e-6)**	**2.77**	**(1.7e-6)**
							
**Overall Significance**		**Array subjects**		**New subjects**		**All subjects**	
		5	**(6.4e-5)**	6	**(2.8e-6)**	6	**(2.8e-6)**

Column 2: average MFS/UC ratio from array experiments. Remaining columns: ratios of geometric mean transcript abundances of MFS affected *vs *unaffected control for three subject groups, and Wilcoxon p values for the null hypothesis of no between group differences. The three groups are: (1) "Array Subjects": 30 of the original 36 array subjects (16 known *FBN1 *mutations and 14 unaffected controls); (2) "New Subjects": 32 new subjects including 16 MFS based on clinical criteria and 16 unaffected controls; and (3) "All Subjects": 42 MFS affected compared to 32 unaffected (all of the above groups plus 10 additional characterized *FBN1 *mutations and 2 additional controls). (See Table 1 for subject details.) Bold: array validation or significant p value (p < 0.05). Italic: significant p-value but difference opposite to the expected direction. N/A: see text. The bottom section of the table provides a summative assessment: the (binomial) p-value of the observed number of significant qRT-PCR validations (p < 0.05) under the null hypothesis of no between-group differences.

It is noteworthy that the expression values in the qRT-PCR assays do not appear to be normally distributed. E.g., box-and-whisker plots of normally distributed data analogous to Figure 2 would be expected to show less than 1% of the samples as "outliers" (the triangles plotted outside the "whisker" ranges, which encompass ± 2.7 times the estimated standard deviation), whereas Figure 2 has 3.7 times as many outliers among the UC samples and 9.4 times as many among MFS samples (also see Table 5). In response to this apparent non-normality, we used the non-parametric Wilcoxon rank sum test to evaluate the significance of gene expression differences between groups, a more conservative choice than the usual t-test in such a circumstance. Under the null hypothesis that data for the two groups are sampled from the same (unspecified) continuous distribution, the test is sensitive to a shift in the location (e.g. mean and median) of the distributions (but not necessarily sensitive to a shift in variance, as seen in most genes here).

Table 4 shows that when all of the samples are combined, 6 of 10 genes were validated. Under the null hypothesis that the genes are independent and not influenced by MFS status, the chance that the qRT-PCR results would confirm 6 of 10 predictions as being statistically significant (Wilcoxon p value < 0.05) is less than 2.8 × 10^-6^. Fold changes and Wilcoxon p values for all 10 genes are shown in Table 4 based on all 74 subjects and separately, on 30 of the original 36 array subjects and 32 new subjects (Table 1). Among 10 genes selected based on the array experiments, 8 show consistent direction of difference by qRT-PCR on the same array subjects, and 5 of these 8 genes are statistically significant. Similarly, 6 of these 10 genes show the same direction, and are significant, when tested on new subjects, as are 6 of 10 when tested on all subjects. Four of the 10 genes are significant in all three sets of subjects showing that the array experiments were effective in detecting MFS associated genes.

The two genes marked N/A show marginally statistically significant changes in Array Subjects but very highly significant changes in the opposite direction in New Subjects. One of these genes is Fibrillin-1, the key gene implicated in MFS. In our array experiments, *FBN1 *was significantly repressed (1.89 fold, q = 2.52 × 10^-10^; Table 4) and qRT-PCR confirmed that its (geometric) mean level was 1.47 fold lower in MFS based on (predominantly) the same subjects (p = 0.052, Table 5 "Array Subjects"). However, qRT-PCR shows that in "New Subjects" this gene is significantly more highly expressed in MFS than controls (2.71 fold higher, p = 3.3 × 10^-9^). Viewed another way, over all subjects, the difference in mean *FBN1 *levels between affected and unaffected subjects is not statistically significant, but 22 of 42 MFS subjects have values more extreme than the most extreme value in the unaffected samples (9 lower, 13 higher). It seems likely that MFS subjects exhibit considerable heterogeneity in *FBN1 *mRNA levels and our original and new subject populations may not be equivalent samples. As was previously shown, nonsense mutations lead to nonsense-mediated decay of the mutant mRNA and reduced fibrillin protein synthesis [26-28]. On the other hand, all the known *FBN1 *missense mutations used in this study are cysteine substitutions in calcium-binding EGF domains. *FBN1 *transcript levels and fibrillin protein synthesis were normal in these cells [8,26,27]. As the distribution of mutations types in the second set of clinically identified MFS subjects is unknown, they may not replicate the distribution of the two mutation classes in the original MFS subjects. An additional factor is a difference in cell culture passage number. Nearly all the UC and all new MFS subjects were used at passage 2 while the original MFS cultures were used between passage 3 and 6. The second gene marked N/A in Table 4 is procollagen C-endopeptidase (*PCOLCE*), another gene with a clear mechanistic role in maintenance of the extracellular matrix [28]. It is plausibly co-regulated with *FBN1*, showing a moderately strong correlation to *FBN1 *in our qRT-PCR data. Given these uncertainties as to the homogeneity of our subject population with respect to these two genes, we considered that overall ratios and p-values were potentially misleading; hence we chose to omit them.

A surprising result of the qRT-PCR measurements, evident in Figure 2 and analyzed in more detail in Table 5, are the variability of expression of these genes, both in unaffected and especially in MFS subjects. We looked at both the full range of the data and at the interquartile range (IQR; the range between the 25^th ^and 75^th ^percentiles, quantifying the spread in the central majority of the population, a statistic that is very robust to the influence of outliers). In general, most genes in UC samples show interquartile ranges of at most 3-fold, with two showing roughly 4-fold changes (*LMO7, VDR*). IQR for MFS samples is similar, with a slight tendency to higher variability (e.g., higher mean and quartiles, and 6 of 10 showing greater variability across MFS samples than UC) and a few dramatic exceptions (*VDR *11 fold; *ELN *24 fold). Variability is more pronounced over the full range. In the UC samples the majority vary by 16–230 fold, and one by more than 1000 fold. MFS subjects show greater variability than UC samples in at least 8 of the 10 genes. The least variable gene (*PDCD10*) changes by more than 64-fold across MFS subjects, the majority vary by more than 225-fold, with some (*ELN *and *INHBA*) showing subject-to-subject differences exceeding 1600-fold. Controlling for age, sex, or missense/nonsense mutations did not reduce the marked variability. (The effect of cell passage number (see Methods) could not be tested.)

**Table 5:** Gene variability by qRT-PCR

	**Interquartile range**			**Full range**			**# Outliers**	
**Gene**	**UC**	**MFS**	**MFS/UC**	**UC**	**MFS**	**MFS/UC**	**UC**	**MFS**

*COL1A2*	2.9	1.9	0.7	19.2	66.1	3.4	0	6
*ELN*	2.4	24.3	10.4	1190.4	1687.3	1.4	4	0
*FBN1*	1.6	3.3	2.0	4.4	69.3	15.6	0	2
*INHBA*	2.2	3.7	1.7	124.5	2218.2	17.8	4	3
*LMO7*	4.7	2.0	0.4	25.9	141.1	5.4	0	7
*PCOLCE*	2.6	1.8	0.7	5.9	295.5	50.3	0	5
*PDCD10*	1.8	1.4	0.8	103.6	64.7	0.6	1	6
*PLOD2*	2.0	3.1	1.5	16.0	251.2	15.7	1	3
*PTGES*	2.8	3.7	1.3	31.7	225.0	7.1	1	1
*VDR*	3.9	11.3	2.9	230.3	233.0	1.0	1	0
min	1.6	1.4	0.4	4.4	64.7	0.6	0.0	0.0
median	2.5	3.2	1.4	28.8	229.0	6.3	1.0	3.0
mean	2.7	5.7	2.2	175.2	525.1	11.8	1.2	3.3
max	4.7	24.3	10.4	1190.4	2218.2	50.3	4.0	7.0
# > 1			6			8		

For the 10 genes validated by qRT-PCR, we summarize variability across 32 UC and 42 MFS samples. Interquartile Range: fold change between the 25^th ^and 75^th ^percentiles. Full Range: fold change between the most extreme pair of samples. #Outliers: number of outliers (triangles in Figure 2). If the data were normally distributed, approximately 1% of the points would so identified, i.e., on average 0.32 per gene for UC and 0.42 per gene for MFS samples.

For the long term, this variability emphasizes the importance of studies involving carefully targeted and/or large subject populations, and of developing simple assays for relevant phenotypic predictors, since small samples may be non-representative. For the purposes of the present study, variability complicates interpretation of the results for similar reasons. For example, in our previous discussion of *FBN1 *we remarked that it did not meet our criterion for a statistically significant shift in expression level in the "All Subjects" grouping, although it remains likely that *FBN1 *level is influenced by MFS status and specific types of *FBN1 *mutations. Similar remarks apply to other genes, where increased variability across MFS subjects suggests these genes are involved in disease processes, but not by a mechanism that consistently elevates or consistently represses expression. These large variations in expression may limit the extent of our interpretations and will require more focused studies and/or additional statistical power before an association with the MFS phenotype can be established firmly. Alternatively the expression distribution might be influenced by other factors such as the genotypes of *FBN1 *or disease modifying genes that we have not yet identified.

## Discussion

We conducted a small-scale gene expression analysis in MFS affected subjects. We hypothesized that subsets of the expression phenotype might indicate genetic and epigenetic factors that are causal or predictive of the MFS diseased state, and more generally, of the aneurysmal state. To conduct this test, we hypothesized that primary cultures of skin fibroblasts derived from punch biopsies could be a simple, robust model. An important advantage to using this system is the ability to collect samples from large numbers of both affected and unaffected subjects. Fibroblasts express *FBN1*, and distinct fibrillin protein phenotypes have been identified in fibroblast cultures from MFS individuals [26,27,29]. The protein profiles have been linked to distinct classes of *FBN*1 mutations and clinical phenotypes [8,30].

We used spotted membrane DNA array screening to identify gene expression changes associated with *FBN1 *mutations. The most important outcome of this study is the identification of a small number of differentially expressed genes that distinguished skin fibroblasts of MFS affected individuals from the fibroblasts of unaffected controls. Our DNA array experiments identified a subset of genes that were validated in a new group of subjects. Due to the size of our study we chose to focus on the expression pattern of affected vs. unaffected and chose 10 genes for validation.

We can speculate on how a number of the validated genes could contribute to the MFS phenotype in a mechanistic way. MFS is a heritable fibrillinopathy where defects in the synthesis and/or assembly of fibrillin-1 microfibrils lead to impairment in the elastic fibrils that confer resilience and recoil in elastic tissues [31,32]. For over a decade it was thought that the microfibrillar component of elastic fibers provided a three-dimensional scaffold for the assembly of elastin in the process of elastogenesis [33]. The histopathological abnormalities of aneurysmal vessels include abnormal extracellular matrix protein accumulation, fragmentation and disorganization of the elastic fibers in the medial layer of the vascular wall, and a generalized loss of elastin content [31,32]. Analysis of mouse models of fibrillin-1 deficiency suggested that the primary defect is not in elastogenesis but rather in elastic fiber homeostasis [34]. Characterization of the mouse model led to a model of acquired elastolysis [35] with evidence suggesting that the altered elastic fiber structure could change the expression phenotype of the underlying vascular smooth muscle cells, resulting in the increased expression of several proteases. A number of investigators have identified the association of specific types of *FBN1 *mutations with an increased susceptibility to protease digestion [9] and with the ability of *FBN1 *fragments to up regulate protease expression in culture [36]. In contrast, our study identifies elastin as one of the most significantly regulated genes in the affected cell lines. Surprisingly, elastin mRNA is markedly decreased (Table 4; 15.0^-1^, p = 1.6 × 10^-8^) suggesting a very early defect in elastogenesis that starts at the level of elastin transcription or the regulation of elastin message stability. Interestingly, the vitamin D receptor was also significantly decreased in MFS subjects (Table 4; 2.5^-1^, p = 7.3 × 10^-3^). Vitamin D is known to decrease elastin mRNA by decreasing message stability in cultured cells [37]. Vitamin D treatment also is known to decrease both elastin content and the number of elastic lamellae in the aortas of animals treated pre and post-natally [38].

Recent publications have also described an increasing role for transforming growth factor beta (TGF-beta) in the expression of the MFS phenotype. Mutations in both *TGF-beta *receptors have been identified in humans with heritable forms of aneurysm [39-41]. In a mouse model of MFS the mutant phenotype includes developmental abnormalities of the distal alveolae of the lung, associated with increased TGF-beta protein and activity [42,43]. Our arrays did not detect any significant difference in TGF beta expression level, while a related family member, *INHBA*, was not validated by the qRT-PCR analysis. More recently, TGF-beta antagonists, including a neutralizing antibody to TGF-beta and losartan (an angiotensin II type receptor ATI blocker) were able to prevent and reverse aneurysmal progression in the same mouse model [44]. We found significant differences in the expression of a number of genes in this pathway including *VDR *(Table 4; 2.53^-1^, p = 7.3 × 10^-3^) and *TSC2 *(Table 2; 1.64, q = 1.3 × 10^-4^). The vitamin D receptor (*VDR*) is a negative regulator of TGF-beta transcriptional activation [45] and as mentioned above is significantly decreased in MFS skin fibroblasts. In contrast, the tuberous sclerosis complex 2 (*TSC2*), a potent activator of TGF-beta transcription, is expressed more highly in MFS fibroblasts (Table 2; 1.64, q = 1.3 × 10^-4^). Another gene, *LMO7 *(a protein that connects the nectin-afadin and E-cadherin-catenin systems through alpha-actinin and therefore regulates cell adhesion) [46] is induced by TGF-beta and is significantly elevated in MFS fibroblasts (Table 4; 2.77, p = 1.7 × 10^-6^). The behavior of all three genes is consistent with enhanced TGF-beta activity that could contribute to the aneurysmal phenotype if smooth muscle cell loss or differentiated phenotype contributes to the pathological process.

Our data also identify several matrix metalloproteases, including *ADAM12 *(Table 2; 1.65^-1^, q = 5.1 × 10^-9^), *MMP1 *(1.14, q = 2.1 × 10^-2^) and the metalloproteinase inhibitor *TIMP3 *(1.39^-1^, q = 6.0 × 10^-6^), with significant differences in expression between the unaffected and affected group. Several collagens including *COL 3A1 *(Table 2; 2.41^-1^, q = 2.1 × 10^-4^), *COL1A2 *(Table 4; 2.27^-1^, p = 1.4 × 10^-4^) and the lysine hydroxylase *PLOD2 *(Table 4; 1.85, p = 2.7 × 10^-4^) (which forms hydroxylysine residues in collagen which serve as attachment sites for carbohydrates that contribute to the stability of the intermolecular collagen cross-links) are also significantly changed. The appearance of these genes suggests a broad change in the extracellular fibrillar structure and composition in affected cells. Many of these matrix proteins and modifying enzymes are regulated by TGF-beta. [47,44,48].

*PDCD10 *(programmed cell death gene 10) is increased in the MFS samples (Table 4; 1.41, p = 5.0 × 10^-5^). *PDCD10 *was recently identified as one of three genes responsible for a heritable form of cerebral cavernous malformations (CCM3) characterized by abnormally enlarged capillary cavities causing seizures and hemorrhages [49]. These vascular malformations appear to result from a failure in vascular morphogenesis and/or remodeling. In CCM, the endothelial tubes continue to expand and stabilizing pericytes are not recruited. The result is a vessel with an enormously dilated lumen and increased fragility [50].

When we initiated these studies we had two important concerns. First, *a priori*, it is not obvious that cultured skin fibroblasts would exhibit significant expression differences for other genes in MFS subjects. However, the skin is one of the connective tissues affected in MFS, and our experiments confirm a significant MFS expression phenotype in cultured fibroblasts. The second concern is that these differences may not be relevant to aneurysmal disease, believed to be largely a disease of smooth muscle cells. Obviously, we cannot be sure that the gene changes discovered in fibroblasts are the same gene changes found in aortic smooth muscle cells, but, given that the two cell types share commonalities in their morphology, extracellular matrix environment and the common fibrillin defect, it is reasonable to suspect that they also share some relevant core changes in fibrillin-related pathways. While our fibroblast study cannot answer this question, it did identify some differentially expressed genes whose known roles are suggestive of involvement in SMC tissue failure. A third concern arose during the course of the study – namely, the large sample-to-sample variation in gene expression levels. This variability needs to be recognized as an important confounding factor in expression analysis studies. It also supports our decision to use skin fibroblasts rather than aortic SMC as our sample source.

## Conclusion

Our ultimate goal is to use genome-wide expression analysis to identify and classify people at risk for developing aneurysms of the ascending thoracic aorta. Our preliminary data support the idea that there are common mechanisms triggered by mutations in *FBN1 *that lead to identifiable differences in gene expression between unaffected control and MFS affected cultured fibroblasts. Some of these changes appear to be downstream from TGF-beta activation. While the limited phenotype described here is only a partial description of the potential "mutation associated" expression profile, the results suggest that a complete genomic screen of several hundred MFS vs. unaffected cell lines, using *FBN1 *mutation detection, microarray expression analysis and qRT-PCR validation of possible biomarkers, could lead to the identification of genes that contribute to the mechanistic events that initiate vessel wall destruction.

## Methods

### Cell culture, RNA isolation, and array hybridization

Study participants were recruited under an institutional review board approved protocol and informed consent. A skin punch biopsy was used to establish a fibroblast cell culture. Unaffected control subjects were selected from patients visiting dermatology clinics. All of the cell strains (in the array group, between passage 2 and 6 and in the New Subject group, passage 2) were grown to confluence in SmGM2 +10% FBS + bullet kit containing recombinant EGF, FGF and insulin (BioWhittaker). After reaching confluence, the medium was modified to include ascorbic acid at 50-μg/ml [51]. Fresh medium was added after 24 hours. At 48 h, total RNA was isolated using a guanidinium iso-thiocyanate-phenol-chloroform extraction protocol [52]. Each sample was analyzed for quality and quantity by UV spectroscopy and gel electrophoresis. For hybridization to individual arrays, 1 μg of total RNA was used to synthesize ^33^P-dCTP labeled first strand cDNA with Invitrogen Superscript II. Generally, each sample was labeled twice and hybridized to duplicate Research Genetics GF211 arrays (4 arrays in all). Each array was hybridized with 30–60 million cpm of probe for 18 h then washed extensively at 50°C in 0.5 × SSC +0.1% SDS. Multiple exposures were collected on a Storm phosphorimager. Images were imported using Research Genetics Pathways 3 software, and processed as described in "Array Data Normalization."

### Quantitative RT-PCR

Validation of the array results was done using Applied Biosystems pre-designed qRT-PCR primer and probe sets (PDAR's, see ABI P/N 4333458_a.pdf for the complete protocol). These assays use a standard PCR format where all the assays are performed with the same PCR cycling conditions. Quantification is based on calculating a ratio to an included calibrator sample. Our calibrator was 50% Stratagene Universal Target total RNA and 50% total RNA pooled from the 36 array subjects listed in Table 1. We synthesized first strand cDNA using random primers and the ABI High Capacity cDNA Archive Kit (P/N 4322171). Duplicate PCR reactions were set up in a final volume of 25 ul using 10 ng equivalents of input total RNA. We used the ABI 7900 machine with the standard cycling protocol, increasing the number of cycles to 40. *GUSB *and *TBP *were used as an internal reference in all of the samples and calibrator. The delta delta Ct method [54] was used to calculate ratios of each gene in each sample against the calibrator RNA. Both references gave similar results and we present the G*USB *quantification.

### Array data normalization

Normalization refers to the process of attempting to correct for the many sources of systematic variation in cDNA array. In outline, we do the following. The median of log_10_-transformed expression values for each gene across all exposures of all arrays is calculated, and serves as a common baseline for comparison between experiments. Then, for each exposure, we compute a smooth "local" (loess) regression line between its log intensities and the median levels. The resulting regression function transforms the expression values of each exposure to the scale of the common baseline while capturing nonlinear effects. Finally, we combine data from multiple exposures of an array by taking their medians. This technique builds on earlier work [54,55] and more details are reported in [56]. One innovation introduced here is the following: to reduce the impact of outliers and differentially expressed genes on the inferred levels of other genes, the loess regression is based on only a "stable" subset of genes uniformly chosen across all intensity levels. Specifically, for each gene we calculate the rank of its measured intensity in each exposure, and the median absolute deviation (MAD) of its rank across exposures. We sort all n genes by increasing median intensity and partition them into n/5 groups of consecutive genes. From each group of 5, the gene with the minimum MAD statistic is chosen. This selection of n/5 genes comprises the stable subset of genes on which our regression is based. Other approaches to normalization based on stable genes have been proposed [57,58] but those techniques seem more complex, and choose stable genes in ways that potentially yield sparse coverage of some regions of the intensity spectrum, consequently increasing the influence of anomalous or differentially expressed genes on the normalization in those regions. As intensity-dependent non-linearity is a key concern in our normalization, uniform representation of intensities in the stable subset is valuable.

Correlation analyses and ANOVA tests identified possible technical biases in a subset of hybridizations. As a consequence, hybridization data from seven Marfan and two unaffected samples were eliminated from further study (indicated by blanks in the platform columns in Table 1). Most of these subjects were included in the qRT-PCR analysis and appear in the "All Subjects" category of Table 4.

### Differential expression and permutation analysis

We used standard t-test p-values to rank genes for evidence of differential expression, coupled with FDR analysis, and selected a conservative q = .001 cutoff threshold. We chose the classical t-test for its familiarity and simplicity. The non-parametric Wilcoxon rank sum test yielded very similar gene rankings. We decided against the commonly used SAM-statistic [59] because of an interest in genes with low expression levels. SAM is biased somewhat against such genes [60].)

To avoid dependence on parametric assumptions about the array data, we performed a permutation test to determine if the large number of significant genes we found could easily have arisen by chance. For a comparison of subject groups A and B, we combined A and B and then randomly split the combined group into two groups A' and B', such that groups A and A' are the same size, groups B and B' are the same size, and the technical replicates of any one sample are either all in A' or all in B'. Hence, the disease status labels were randomly permuted to determine whether the true disease was responsible for the observed result of 283 genes with q < 0.001. We then computed the number of differentially expressed genes at the same cutoff threshold (FDR < 0.001) in each such random partition.

### qRT-PCR data analysis

As part of the statistical analysis of the qRT-PCR data, we performed per-gene regression tests for age, sex and/or group effects on expression levels (in addition to MFS status). There were no significant sex effects in any of the tests. Age effects were marginally significant for two genes, but not after Bonferroni correction, and furthermore would not have altered the significance of MFS status even if included. For some genes, inclusion of subject in "new" vs. "original" subject groups (Table 1) was significant, but again correction for this effect did not alter the significance of MFS status. Hence, we have chosen to present results only for the simplest model, which does not attempt to adjust for any of these covariates.

Significance of differential expression was quantified by Wilcoxon rank sum test; see Figure 2, Table 4. "Overall significance" results reported in the last line of Table 4 follow from a simple binomial model.

## Abbreviations

MFS- Marfan Syndrome.

MS- Missense mutation.

NS- Nonsense mutation.

UC- Unaffected Control.

qRT-PCR- Quantitative Real Time Polymerase Chain Reaction.

FDR- False Discovery Rate.

SAM- Statistical Analysis of Microarrays.

IQR- Interquartile Range.

MAD- median absolute deviation.

PDAR- Pre-designed Assay Reagents.

PTC  –  Premature termination codon.

Del Ex – Deleted exon(s).

## Authors' contributions

EM conceived of and designed the study. CZM and EM performed all of the experiments. ZY, JCJ, WLR and ME performed all the statistical analysis, UF and DM contributed the Marfan skin fibroblasts with known mutations, and DM contributed most of the normal skin fibroblasts. SMS contributed to the design of the study. WLR, ERM and SMS are co-senior authors. All of the authors contributed to the writing of the manuscript.

## Supplementary Material

Additional file 1 Statistical analysis of the normalized array data. The data provided represent the statistical analysis of the normalized data from the array subjects identified (A) in Table 1. Click here for file

Additional file 2 Delta delta CT values for the qRT-PCR analysis. The data provide the average delta delta CT values for all the subjects identified (T) in Table 1. Click here for file

## References

[B1] Coady MA, Rizzo JA, Goldstein LJ, Elefteriades JA (1999). Natural history, pathogenesis, and etiology of thoracic aortic aneurysms and dissections. Cardiol Clin.

[B2] Januzzi JL, Marayati F, Mehta RH, Cooper JV, O'Gara PT, Sechtem U, Bossone E, Evangelista A, Oh JK, Nienaber CA, Eagle KA, Isselbacher EM (2004). Comparison of aortic dissection in patients with and without Marfan's syndrome (results from the International Registry of Aortic Dissection). Am J Cardiol.

[B3] Pyeritz RE (2000). The Marfan syndrome. Annu Rev Med.

[B4] Dietz HC, Cutting GR, Pyeritz RE, Maslen CL, Sakai LY, Corson GM, Puffenberger EG, Hamosh A, Nanthakumar EJ, Curristin SM, . (1991). Marfan syndrome caused by a recurrent de novo missense mutation in the fibrillin gene. Nature.

[B5] Dietz HC, Pyeritz RE, Hall BD, Cadle RG, Hamosh A, Schwartz J, Meyers DA, Francomano CA (1991). The Marfan syndrome locus: confirmation of assignment to chromosome 15 and identification of tightly linked markers at 15q15-q21.3. Genomics.

[B6] Collod-Beroud G, Le Bourdelles S, Ades L, Ala-Kokko L, Booms P, Boxer M, Child A, Comeglio P, de Paepe A, Hyland JC, Holman K, Kaitila I, Loeys B, Matyas G, Nuytinck L, Peltonen L, Rantamaki T, Robinson P, Steinmann B, Junien C, Beroud C, Boileau C (2003). Update of the UMD-FBN1 mutation database and creation of an FBN1 polymorphism database. Hum Mutat.

[B7] Loeys B, De Backer J, Van Acker P, Wettinck K, Pals G, Nuytinck L, Coucke P, de Paepe A (2004). Comprehensive molecular screening of the FBN1 gene favors locus homogeneity of classical Marfan syndrome. Hum Mutat.

[B8] Schrijver I, Liu W, Brenn T, Furthmayr H, Francke U (1999). Cysteine Substitutions in Epidermal Growth Factor-Like Domains of Fibrillin-1: Distinct Effects on Biochemical and Clinical Phenotypes. Am J Hum Genet.

[B9] Robinson PN, Booms P, Katzke S, Ladewig M, Neumann L, Palz M, Pregla R, Tiecke F, Rosenberg T (2002). Mutations of FBN1 and genotype-phenotype correlations in Marfan syndrome and related fibrillinopathies. Hum Mutat.

[B10] Gabbiani G (2003). The myofibroblast in wound healing and fibrocontractive diseases. J Pathol.

[B11] Desmouliere A, Chaponnier C, Gabbiani G (2005). Tissue repair, contraction, and the myofibroblast. Wound Repair Regen.

[B12] Koumas L, Smith TJ, Feldon S, Blumberg N, Phipps RP (2003). Thy-1 expression in human fibroblast subsets defines myofibroblastic or lipofibroblastic phenotypes. Am J Pathol.

[B13] Mariadason JM, Arango D, Augenlicht LH (2004). Customizing chemotherapy for colon cancer: the potential of gene expression profiling. Drug Resist Updat.

[B14] Ramaswamy S, Tamayo P, Rifkin R, Mukherjee S, Yeang CH, Angelo M, Ladd C, Reich M, Latulippe E, Mesirov JP, Poggio T, Gerald W, Loda M, Lander ES, Golub TR (2001). Multiclass cancer diagnosis using tumor gene expression signatures. Proc Natl Acad Sci U S A.

[B15] van de Vijver MJ, He YD, van't Veer LJ, Dai H, Hart AA, Voskuil DW, Schreiber GJ, Peterse JL, Roberts C, Marton MJ, Parrish M, Atsma D, Witteveen A, Glas A, Delahaye L, van, Bartelink H, Rodenhuis S, Rutgers ET, Friend SH, Bernards R (2002). A gene-expression signature as a predictor of survival in breast cancer. N Engl J Med.

[B16] Kote-Jarai Z, Williams RD, Cattini N, Copeland M, Giddings I, Wooster R, tePoele RH, Workman P, Gusterson B, Peacock J, Gui G, Campbell C, Eeles R (2004). Gene expression profiling after radiation-induced DNA damage is strongly predictive of BRCA1 mutation carrier status. Clin Cancer Res.

[B17] Khan J, Wei JS, Ringner M, Saal LH, Ladanyi M, Westermann F, Berthold F, Schwab M, Antonescu CR, Peterson C, Meltzer PS (2001). Classification and diagnostic prediction of cancers using gene expression profiling and artificial neural networks. Nat Med.

[B18] Lee YF, John M, Falconer A, Edwards S, Clark J, Flohr P, Roe T, Wang R, Shipley J, Grimer RJ, Mangham DC, Thomas JM, Fisher C, Judson I, Cooper CS (2004). A gene expression signature associated with metastatic outcome in human leiomyosarcomas. Cancer Res.

[B19] Ebert BL, Golub TR (2004). Genomic approaches to hematologic malignancies. Blood.

[B20] Tan PK, Downey TJ, Spitznagel EL, Xu P, Fu D, Dimitrov DS, Lempicki RA, Raaka BM, Cam MC (2003). Evaluation of gene expression measurements from commercial microarray platforms. Nucleic Acids Res.

[B21] Lee JK, Bussey KJ, Gwadry FG, Reinhold W, Riddick G, Pelletier SL, Nishizuka S, Szakacs G, Annereau JP, Shankavaram U, Lababidi S, Smith LH, Gottesman MM, Weinstein JN (2003). Comparing cDNA and oligonucleotide array data: concordance of gene expression across platforms for the NCI-60 cancer cells. Genome Biol.

[B22] Jarvinen AK, Hautaniemi S, Edgren H, Auvinen P, Saarela J, Kallioniemi OP, Monni O (2004). Are data from different gene expression microarray platforms comparable?. Genomics.

[B23] Kuo WP, Kim EY, Trimarchi J, Jenssen TK, Vinterbo SA, Ohno-Machado L (2004). A primer on gene expression and microarrays for machine learning researchers. J Biomed Inform.

[B24] Bullinger L, Dohner K, Bair E, Frohling S, Schlenk RF, Tibshirani R, Dohner H, Pollack JR (2004). Use of gene-expression profiling to identify prognostic subclasses in adult acute myeloid leukemia. N Engl J Med.

[B25] Storey JD (2002). A direct approach to false discovery rates. Jorunal of the Royal Statistical Society.

[B26] Aoyama T, Francke U, Dietz HC, Furthmayr H (1994). Quantitative differences in biosynthesis and extracellular deposition of fibrillin in cultured fibroblasts distinguish five groups of Marfan syndrome patients and suggest distinct pathogenetic mechanisms. J Clin Invest.

[B27] Brenn T, Aoyama T, Francke U, Furthmayr H (1996). Dermal fibroblast culture as a model system for studies of fibrillin assembly and pathogenetic mechanisms: defects in distinct groups of individuals with Marfan's syndrome. Lab Invest.

[B28] Prockop DJ, Sieron AL, Li SW (1998). Procollagen N-proteinase and procollagen C-proteinase. Two unusual metalloproteinases that are essential for procollagen processing probably have important roles in development and cell signaling. Matrix Biol.

[B29] Milewicz DM, Pyeritz RE, Crawford ES, Byers PH (1992). Marfan syndrome: defective synthesis, secretion, and extracellular matrix formation of fibrillin by cultured dermal fibroblasts. J Clin Invest.

[B30] Schrijver I, Liu W, Odom R, Brenn T, Oefner P, Furthmayr H, Francke U (2002). Premature termination mutations in FBN1: distinct effects on differential allelic expression and on protein and clinical phenotypes. Am J Hum Genet.

[B31] Milewicz DM, Urban Z, Boyd C (2000). Genetic disorders of the elastic fiber system. Matrix Biol.

[B32] Urban Z, Boyd CD (2000). Elastic-fiber pathologies: primary defects in assembly-and secondary disorders in transport and delivery. Am J Hum Genet.

[B33] Robert L (1996). Aging of the vascular wall and atherogenesis: role of the elastin- laminin receptor [published erratum appears in Atherosclerosis 1996 Sep 27;126(1):173]. Atherosclerosis.

[B34] Pereira L, Lee SY, Gayraud B, Andrikopoulos K, Shapiro SD, Bunton T, Giery NJ, Dietz HC, Sakai LY, Ramirez F (1999). Pathogenetic sequence for aneurysm revealed in mice underexpressing fibrillin-1. Proc Natl Acad Sci U S A.

[B35] Bunton TE, Biery NJ, Myers L, Gayraud B, Ramirez F, Dietz HC (2001). Phenotypic alteration of vascular smooth muscle cells precedes elastolysis in a mouse model of Marfan syndrome. Circ Res.

[B36] Booms P, Pregla R, Ney A, Barthel F, Reinhardt DP, Pletschacher A, Mundlos S, Robinson PN (2005). RGD-containing fibrillin-1 fragments upregulate matrix metalloproteinase expression in cell culture: a potential factor in the pathogenesis of the Marfan syndrome. Hum Genet.

[B37] Pierce RA, Kolodziej ME, Parks WC (1992). 1,25-Dihydroxyvitamin D3 represses tropoelastin expression by a posttranscriptional mechanism. J Biol Chem.

[B38] Norman P, Moss I, Sian M, Gosling M, Powell J (2002). Maternal and postnatal vitamin D ingestion influences rat aortic structure, function and elastin content. Cardiovasc Res.

[B39] Loeys BL, Chen J, Neptune ER, Judge DP, Podowski M, Holm T, Meyers J, Leitch CC, Katsanis N, Sharifi N, Xu FL, Myers LA, Spevak PJ, Cameron DE, De Backer J, Hellemans J, Chen Y, Davis EC, Webb CL, Kress W, Coucke P, Rifkin DB, De Paepe AM, Dietz HC (2005). A syndrome of altered cardiovascular, craniofacial, neurocognitive and skeletal development caused by mutations in TGFBR1 or TGFBR2. Nat Genet.

[B40] Loeys S (2006). N Engl J Med.

[B41] Mizuguchi T, Collod-Beroud G, Akiyama T, Abifadel M, Harada N, Morisaki T, Allard D, Varret M, Claustres M, Morisaki H, Ihara M, Kinoshita A, Yoshiura K, Junien C, Kajii T, Jondeau G, Ohta T, Kishino T, Furukawa Y, Nakamura Y, Niikawa N, Boileau C, Matsumoto N (2004). Heterozygous TGFBR2 mutations in Marfan syndrome. Nat Genet.

[B42] Neptune ER, Frischmeyer PA, Arking DE, Myers L, Bunton TE, Gayraud B, Ramirez F, Sakai LY, Dietz HC (2003). Dysregulation of TGF-beta activation contributes to pathogenesis in Marfan syndrome. Nat Genet.

[B43] Ng CM, Cheng A, Myers LA, Martinez-Murillo F, Jie C, Bedja D, Gabrielson KL, Hausladen JM, Mecham RP, Judge DP, Dietz HC (2004). TGF-beta-dependent pathogenesis of mitral valve prolapse in a mouse model of Marfan syndrome. J Clin Invest.

[B44] Habashi JP, Judge DP, Holm TM, Cohn RD, Loeys BL, Cooper TK, Myers L, Klein EC, Liu G, Calvi C, Podowski M, Neptune ER, Halushka MK, Bedja D, Gabrielson K, Rifkin DB, Carta L, Ramirez F, Huso DL, Dietz HC (2006). Losartan, an AT1 antagonist, prevents aortic aneurysm in a mouse model of Marfan syndrome. Science.

[B45] Wu Y, Craig TA, Lutz WH, Kumar R (1999). Identification of 1 alpha,25-dihydroxyvitamin D3 response elements in the human transforming growth factor beta 2 gene. Biochemistry.

[B46] Ooshio T, Irie K, Morimoto K, Fukuhara A, Imai T, Takai Y (2004). Involvement of LMO7 in the association of two cell-cell adhesion molecules, nectin and E-cadherin, through afadin and alpha-actinin in epithelial cells. J Biol Chem.

[B47] Le Pabic H, L'Helgoualc'h A, Coutant A, Wewer UM, Baffet G, Clement B, Theret N (2005). Involvement of the serine/threonine p70S6 kinase in TGF-beta1-induced ADAM12 expression in cultured human hepatic stellate cells. J Hepatol.

[B48] Kwak HJ, Park MJ, Cho H, Park CM, Moon SI, Lee HC, Park IC, Kim MS, Rhee CH, Hong SI (2006). Transforming growth factor-beta1 induces tissue inhibitor of metalloproteinase-1 expression via activation of extracellular signal-regulated kinase and Sp1 in human fibrosarcoma cells. Mol Cancer Res.

[B49] Bergametti F, Denier C, Labauge P, Arnoult M, Boetto S, Clanet M, Coubes P, Echenne B, Ibrahim R, Irthum B, Jacquet G, Lonjon M, Moreau JJ, Neau JP, Parker F, Tremoulet M, Tournier-Lasserve E (2005). Mutations within the programmed cell death 10 gene cause cerebral cavernous malformations. Am J Hum Genet.

[B50] Coucouvanis E, Martin GR (1995). Signals for death and survival: a two-step mechanism for cavitation in the vertebrate embryo. Cell.

[B51] Jain MK, Layne MD, Watanabe M, Chin MT, Feinberg MW, Sibinga NE, Hsieh CM, Yet SF, Stemple DL, Lee ME (1998). In vitro system for differentiating pluripotent neural crest cells into smooth muscle cells. J Biol Chem.

[B52] Chomczynski P, Sacchi N (1987). Single-step method of RNA isolation by acid guanidinium thiocyanate-phenol-chloroform extraction. Anal Biochem.

[B53] Lossos IS, Czerwinski DK, Alizadeh AA, Wechser MA, Tibshirani R, Botstein D, Levy R (2004). Prediction of survival in diffuse large-B-cell lymphoma based on the expression of six genes. N Engl J Med.

[B54] Dozmorov I, Galecki A, Chang Y, Krzesicki R, Vergara M, Miller RA (2002). Gene expression profile of long-lived snell dwarf mice. J Gerontol A Biol Sci Med Sci.

[B55] Yang YH, Dudoit S, Luu P, Lin DM, Peng V, Ngai J, Speed TP (2002). Normalization for cDNA microarray data: a robust composite method addressing single and multiple slide systematic variation. Nucleic Acids Res.

[B56] Mulvihill ER, Jaeger J, Sengupta R, Ruzzo WL, Reimer C, Lukito S, Schwartz SM (2004). Atherosclerotic plaque smooth muscle cells have a distinct phenotype. Arterioscler Thromb Vasc Biol.

[B57] Kepler TB, Crosby L, Morgan KT (2002). Normalization and analysis of DNA microarray data by self-consistency and local regression. Genome Biol.

[B58] Schadt EE, Li C, Ellis B, Wong WH (2001). Feature extraction and normalization algorithms for high-density oligonucleotide gene expression array data. J Cell Biochem Suppl.

[B59] Tusher VG, Tibshirani R, Chu G (2001). Significance analysis of microarrays applied to the ionizing radiation response. Proc Natl Acad Sci U S A.

[B60] Storey JD, Tibshirani R (2003). Statistical methods for identifying differentially expressed genes in DNA microarrays. Methods Mol Biol.

[B61] Wang J, Guo K, Wills KN, Walsh K (1997). Rb functions to inhibit apoptosis during myocyte differentiation. Cancer Res.

[B62] Liu W, Schrijver I, Brenn T, Furthmayr H, Francke U (2001). Multi-exon deletions of the FBN1 gene in Marfan syndrome. BMC Med Genet.

[B63] Liu WO, Oefner PJ, Qian C, Odom RS, Francke U (1997). Denaturing HPLC-identified novel FBN1 mutations, polymorphisms, and sequence variants in Marfan syndrome and related connective tissue disorders. Genet Test.

